# A Hybrid Microfluidic Electronic Sensing Platform for Life Science Applications

**DOI:** 10.3390/mi13030425

**Published:** 2022-03-10

**Authors:** Abbas Panahi, Ebrahim Ghafar-Zadeh

**Affiliations:** Biologically Inspired Sensors and Actuators (BioSA) Laboratory, Department of Electrical Engineering and Computer Science, Lassonde School of Engineering, York University, Toronto, ON M3J1P3, Canada

**Keywords:** microfluidics, biosensor, ISFET, field-effect transistor, integrated biosensor, packaging, sensor, electronics

## Abstract

This paper presents a novel hybrid microfluidic electronic sensing platform, featuring an electronic sensor incorporated with a microfluidic structure for life science applications. This sensor with a large sensing area of 0.7 mm^2^ is implemented through a foundry process called Open-Gate Junction FET (OG-JFET). The proposed OG-JFET sensor with a back gate enables the charge by directly introducing the biological and chemical samples on the top of the device. This paper puts forward the design and implementation of a PDMS microfluidic structure integrated with an OG-JFET chip to direct the samples toward the sensing site. At the same time, the sensor’s gain is controlled with a back gate electrical voltage. Herein, we demonstrate and discuss the functionality and applicability of the proposed sensing platform using a chemical solution with different pH values. Additionally, we introduce a mathematical model to describe the charge sensitivity of the OG-JFET sensor. Based on the results, the maximum value of transconductance gain of the sensor is ~1 mA/V at Vgs = 0, which is decreased to ~0.42 mA/V at Vgs = 1, all in Vds = 5. Furthermore, the variation of the back-gate voltage from 1.0 V to 0.0 V increases the sensitivity from ~40 mV/pH to ~55 mV/pH. As per the experimental and simulation results and discussions in this paper, the proposed hybrid microfluidic OG-JFET sensor is a reliable and high-precision measurement platform for various life science and industrial applications.

## 1. Introduction

Electrochemical sensors are being used in various applications, such as the food industry, pharmaceutical, oil and gas, environmental monitoring and biomedical engineering [1,2,3,4]. These sensors promise faster and more sensitive techniques for detecting diseases and hazardous materials [5,6].

A well-established member of electrochemical sensors, the so-called ion-sensitive field-effect transistor (ISFET) sensors have been utilized in different applications for ion/charge detection in chemical solutions. The ISFETs range from carbonaceous FET sensors (e.g., graphene, carbon nanotubes) to 2D materials (e.g., MoS_2_) [7,8,9,10]. Many efforts have been made to develop ISFETs based on technologically flawless silicon-based complementary metal-oxide semiconductors (CMOS) technology, resulting in different sensing structures and topologies [4] offering high-yield productions. They have been successfully verified to detect various diseases, such as malaria, influenza, hepatitis B virus, COVID-19 disease and bacteria-based diseases [4,11]. Immobilizing the surface of ISFETs with different biomolecules gives rise to biological FETs (BioFETs), which could be GEN FET (DNA sensors), Cell FET (for cell analysis), Enzyme FET (for enzymatic reactions analysis), etc. [12,13,14]. Additionally, as the ISFETs are sensitive to ions concentration in liquids, they have also been used directly in pH measurements [4].

The above-mentioned miniaturized sensors need to be incorporated with microfluidics to direct the sample toward sensing sites and prevent the solution from reaching the rest of the platform. Microfluidics technology has been subject to intense research in recent decades, aiming to miniaturize bioprocesses and bioanalyses toward enabling lab-on-a-chip [15,16]. Microfluidics and nanofluidics provide other advantages, such as extremely low reagent consumption, low cost, laminar flow operation, reducing exposure risk to toxic materials, parallelization, portability and excellent versatility in design and engineering [17,18]. Microfluidics integrated biosensors are used for parallel analysis of different biomarkers in human blood [19,20,21,22,23,24]. Additionally, the high-throughput integration of fluidics and biosensors has resulted in the creation of commercialized products, such as Ion Torrent’s genome machine and Illumina’s Miseq, in which integrated biosensing has enabled parallelized analysis [25,26,27].

Today, many papers have reported the integration of microfluidics with electronic sensors. Sensor-integrated microfluidic devices were reported for applications such as DNA hybridization [28,29], polymerase chain reaction (PCR) on-chip [30,31], virus detection [32,33], cancer detection [34,35], circulating tumor cells (CTC) analysis [36,37], exosomes analysis [38,39], nucleic acids analysis [40,41], liquid biopsy-based assays for cancer detection [42]. CMOS-based sensors integration with microfluidic is put forward for cell analysis [43,44], chemical sensing [45], optical biosensing [46], ultraprecise micro pumping [47], 2D nanomaterial detection [48], optofluidics biomedical devices [49]. For CMOS sensors integration with microfluidic, the direct-write microfluidic fabrication process (DWFP) was introduced as a comparatively new rapid technique for creating microfluidics atop an electronic chip [50]. The same DWFP method was used for chemical sensing [51]. More advanced techniques were implemented to integrate continuous microfluidic with capacitive CMOS sensor real-time cell analysis [45].

BioFET sensors have shown great potential in unique charge detection capabilities for many applications [4,52]. They should be integrated with microfluidics for successful prospects in point-of-care devices by taking advantage of unique microfluidics features. Many attempts have been made to incorporate BioFETs with microfluidics so far. A graphene FET integrated with microfluidic has been introduced for femtomolar detection of chlorpyrifos [53]. Digital microfluidics was combined with a FET sensor for bioanalysis in lab-on-a-chip [54]. In another work, to accelerate the process of biomarker detection, a silicon nanowire-based FET structure was integrated with microfluidics for parallelization of cardiac biomarker detection in human blood [55]. Graphene FET was implemented in a planar microfluidic channel for ultrasensitive flow velocity sensing application [56]. Microfluidics enabled continuous flow measurement from an extended-gate FET structure applicable for ion sensing [57]. Integrated BioFET with microfluidics was used for aerosol particle detection in the air, promising such integration potentials for high-throughput air quality measurements [58]. Microfluidics integrated CMOS sensor was used to detect metabolite in blood in which PDMS was used to create multiple channels on the sensor [59]. The same packaging method was utilized to develop a novel COMS-microfluidics platform for prostate cancer detection in blood that promises a rapid and cost-effective bedside platform [60]. Elastomeric microfluidics was integrated with a semiconductor sensor with a novel integration method that has negligible sensitivity to the strain from bending and flexures, promising a novel packaging for wearable sensors [61,62]. BioFETs also showed good sensing capabilities toward packaged graphene-based sensors [63]. Microfluidics integrated floating-gate FET sensor was developed, which put forward continuous microfluidics to detect proteins [64]. Organic FET sensors were also integrated with microfluidics, which encompassed reference electrode in the packaged sensor [65]. Microfluidics was integrated with polymeric organic FET for marine environmental detection with high stability [66].

Despite the abovementioned successful experiments on integrated BioFETs with microfluidics at a laboratory scale, specific integration hindrances exist for FET-based lab-on-a-chip for high-throughput integration. As is evident in the BioFETs structures, they need a reference electrode in solution for their operation, which makes the microfluidic integration and packaging extremely difficult [67,68]. One viable solution might be using the on-chip reference electrodes. Still, it is reported that on-chip microfabricated electrodes are not as stable as Ag/AgCl bulky electrodes that are frequently used in BioFETs experiments due to the inherent noise associated with downscaling of material [69]. An engineering solution to avoid bulky reference electrodes is to design a back-gate structure to control the channel conduction in BioFETs and use it for sensor characterization. Removing the reference electrode on top and placing it on the back creates an open space for running a microfluidic channel on the sensing area.

We introduce a simple 3D printed microfluidic integration scheme for a new semiconductor biosensor called OG-JFET. The sensor is based on the open-gate junction field-effect transistor, which works based on a back-gate structure that enables sensor operation without a reference electrode in solution, such as bulky Ag/AgCl electrodes used in previous sensors. This capability led us to use a simple method to integrate a microfluidic channel on top of the sensor. The microfluidic was developed with PolyJET 3D printing technology, which can deliver tiny samples onto the sensing area. Previous sensors used advanced and complex microfabrication processes for creating the microfluidic part due to its small fluidic size. Nevertheless, the large sensing area provided by our sensor and the back gate allowed us to use 3D printing for creating higher minimum feature sizes as large as 2000 µm in microfluidics. Two molds were used to create 3D microfluidics for creating a chamber on top of the sensing area, different from previous planar microfluidics used for BioFET integration.

The PDMS fluidic cell fabrication process is introduced, which can be molded with PolyJET 3D printing. Furthermore, the sensor (OG-JFET) sensing mechanism is detailed with mathematical modeling and experimental evidence for showing the device’s functionality in delivering samples onto the sensor and performing charge detection in an aqueous solution. The sensor provides a back gate, which makes the integration with microfluidic easier. Furthermore, the sensor works at a low voltage (<0.2 V), essential in developing a low-power hand-held electronic platform for industrial applications.

The organization of this paper is as follows: Section 2 explains the electronics sensor (OG-JFET) mathematical modeling; Section 3 introduces the electrical and fluidic packaging and sensor characterization; and Section 4 is dedicated to results and discussion, which is followed by Section 6.

## 2. Electronic Sensor

### 2.1. Open-Gate Junction Field-Effect Transistor and Microfluidics

**Figure 1:**
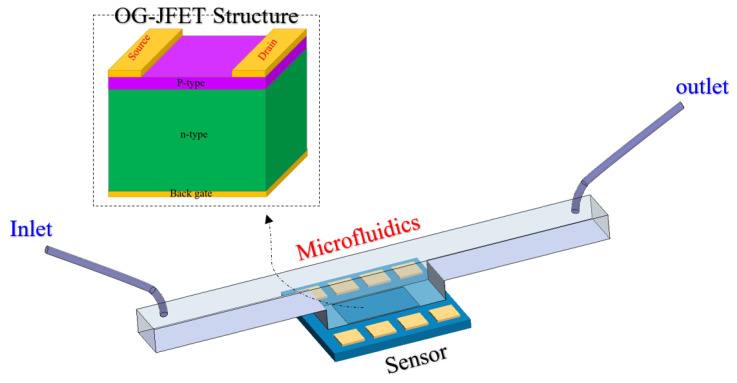
Schematic of OG-JFET structure and the microfluidic chamber.

The current sensing platform is similar to the well-established JFET, with the subtle difference that the top gate of a JFET is removed to open the space for introducing a solution. Physically, the structure is based on a p-type JFET without the top n-type gate, as we reported in our previous works [70,71,72]. Therefore, the structure only has a back gate that can control the conduction channel. The sensor is provided through a standard microfabrication process, which further helps to establish this sensor as a mass-producible platform for industrial applications (for more information about fabrications, we invite the reader to read Refs. [70,71,72]). The platform includes a PDMS channel integrated with the OG-JFET sensor to deliver samples on the sensing area. A schematic of the integrated microfluidic sensor platform is presented in Figure 1.

As shown in Figure 1, the OG-JFET sensor comes with a back gate that is not inside the solution, which helps control the conduction in the channel and can be used as the same reference electrode for voltage or current-based sensing characterization.

The sensor consists of a p-type channel epitaxially grown on a thick n-type layer with doping levels of 5 × 10^15^/cm^3^ and 1 × 10^18^/cm^3^, respectively. The p-type layer thickness is 1.6 µm ± 10% (resistivity of 3.0 Ohm cm ± 10%) on the n-type layer with a thickness of 450 µm with a resistivity of 0.006 to 0.02 Ohm cm. A source and drain with a width of 20 µm are developed on the p-type channel covered with a thick stacked layer of SiO_2_-Si_3_N_4_. The dielectric layers create an encapsulation layer for the drain and source metal layers to avoid contact with the solution.

When the p-type channel is exposed to air or natural moisture, it is expected to have a particular layer of native silicon dioxide on the silicon layer. The native silicon oxide grows naturally when the silicon is open to the air. It is experimentally validated that a layer with a thickness of up to 10 Å can be expected with an exposure of 80 days to solely air [73]. The silicon dioxide provides hydroxyl groups on top of the silicon layer in the open-gate area, which can participate in the chemical reactions with ions in the solution, leading to ion complexation with SiO_2_ surface bonds that create a stacked layer of ions on the surface of silicon [73]. The created ion layers generate a local electric field, which causes a detectable change in the hole conduction inside the p-type layer. The sensor is tested in reverse bias condition of p-n junction for biosensing.

### 2.2. Mathematical Modeling

For a better understanding of sensor operation, mathematical modeling is introduced here, by which the sensing mechanism can be explained in much more detail. The OG-JFET structure can be imagined as half of a p-type JFET (n^+^p JFET) structure in which the two n-type gates are responsible for controlling the sensor. However, in OG-JFET, the top gate is opened to solutions and biomaterials. The n^+^p JFET is called normally on device, which means a voltage should be applied to the gate to close the channel; otherwise, the channel is open, and hole current can be on even if Vg is zero. When a positive voltage is applied on the back gate, the space charge pushes the region to close where the current in the channel reaches zero. This condition is called pinched off state. The pinch-off voltage (Vp) for a n^+^p JFET can be defined with the following equations:

(1) $$V_{p}=V_{p0}-V_{\mathrm{bi}}$$

(2) $$V_{\mathrm{bi}}=V_{t}\ln\left(\frac{N_{a}N_{d}}{n_{i}{}^{2}}\right)$$

(3) $$V_{\mathrm{SD}}\left(\mathrm{sat}\right)=V_{p0}-\left(V_{\mathrm{bi}}+V_{\mathrm{BG}}\right)$$

**Figure 2:**
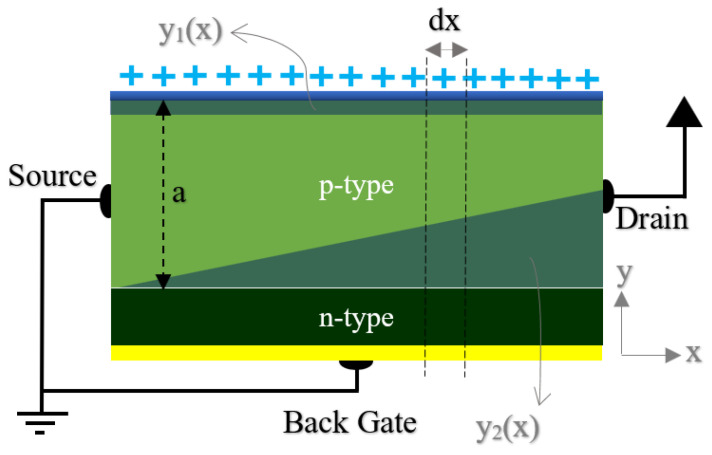
The simplified model of OG-JFET for mathematical modeling of drain–source current.

In Equation (1), the V_p0_ is the inherent pinch-off voltage, which depends on the structure and doping levels. The structure shown in Figure 1 can be simplified for mathematical modeling, as shown in Figure 2.

The Vsd (sat) represents the drain–source voltage, which leads to a pinch-off condition at the drain region [65]. According to Figure 2, considering a differential element of the channel in the x direction, we can develop the current–voltage relation by starting with Ohm’s law and extending the equations based on the space charge effects in the channel.

(4) $$\mathrm{dR}=\frac{\rho \mathrm{dx}}{A\left(x\right)}$$

(5) $$\rho =\frac{1}{{e\mu }_{n}N_{d}}$$

Equation (4) shows Ohm’s law that can be applied to the element of the device shown in Figure 2. The A(x) is the surface perpendicular to the surface. Equation (2) shows the resistivity formula of the device where e is the elemental charge, and µn is the mobility by the assumption of neglecting minor carrier charge. Based on Figure 2, the cross-sectional area is A(x)=[a−y1(x)−y2(x)]W where W is the channel width along the z direction (not shown here). With the assumption of A(x), Equation (4) can be developed to reach (6)

(6) $$I_{D1}\mathrm{dx}={e\mu }_{n}N_{d}W\left[a-x_{1}\left(y\right)-x_{2}\left(y\right)\right]\mathrm{dV}\left(x\right)$$

The depletion width for space charge due to the back gate is

(7) $$x_{2}\left(y\right)={\left[\frac{2\epsilon_{s}\left[V\left(x\right)-V_{\mathrm{bi}}+V_{\mathrm{gs}}\right]}{{\mathrm{eN}}_{d}}\right]}^{\frac{1}{2}}$$

The depletion due to the effect of surface charge on the top gate can be modeled as follows:

(8) $$x_{1}\left(y\right)=\frac{Q}{{\mathrm{qN}}_{A}}t_{\mathrm{ox}}$$

In Equation (8), the Q is the surface charge due to the effects of the aqueous solution, t_ox_ is the thickness of the oxide layer, the N_A_ is the doping level in the semiconductor, and the q is the elemental charge. Due to the surface charge in the solution, a depletion of space charge in the p-type layer occurs (see Figure 2). Equation (8) models that layer. This layer is considered constant along the open-gate area surface, which generates a continual depletion layer thickness in the semiconductor. Therefore, the output value of Equation (8) is a constant value. By substituting Equations (7) and (8) into Equation (6) and integrating over the length of the open-gate area of OG-JFET, the relation for drain–source current Equation (11) can be developed with the following boundary conditions (at 0 and L):

(9) $$w_{1}{}^{2}=\frac{2\varepsilon_{s}}{{\mathrm{qN}}_{A}}\left(V_{\mathrm{bi}}-V_{\mathrm{gs}}\right)\;\;\;\;\;\;\;\;\;\;\;\mathrm{at}\;\mathrm{Lc}=0$$

(10) $$w_{2}{}^{2}=\frac{2\varepsilon_{s}}{{\mathrm{qN}}_{A}}\left(V_{\mathrm{bi}}-V_{\mathrm{gs}}+V_{\mathrm{ds}}\right)\;\mathrm{at}\;\mathrm{Lc}=L$$

The resultant drain–source current relation is (9)

(11) $$I_{D}=\frac{q^{2}{\mu N}_{A}{}^{2}\tau^{3}Z}{\varepsilon_{s}L}\left\{\frac{V_{\mathrm{DS}}}{V_{\mathrm{po}}}-\frac{2}{3}\left(\frac{{\left[\left(V_{\mathrm{bi}}-V_{\mathrm{GS}}+V_{\mathrm{DS}}\right)\right]}^{\frac{3}{2}}}{V_{\mathrm{po}}}-\frac{{\left[\left(V_{\mathrm{bi}}-V_{\mathrm{GS}}\right)\right]}^{\frac{3}{2}}}{V_{\mathrm{po}}}\right)\right\}$$

where $\tau =a-x_{1}\left(y\right)$, with x_1_(y), which demonstrates the depletion effect due to surface charge on the sensor. Equation (11) can be used to calculate gain (g_m_), which is as follows:

(12) $$g_{m}=\frac{\partial I_{D}}{\partial V_{\mathrm{gs}}}$$

It is noteworthy to mention that Equation (11) is valid for the range of voltages $0\leq \left|V_{\mathrm{gs}}\right|\leq V_{\mathrm{po}},\;0\leq \left|V_{\mathrm{ds}}\right|\leq V_{\mathrm{ds}\left(\mathrm{sat}\right)}$.

The sensor structure affects the response of the sensor to change in the charges on the surface (open-gate area). The sensor’s output is current, and it changes with the electric field generated due to the charge on the sensor. The back gate can be used to modulate the drain–source current. Therefore, the charge on top of the sensor can also be considered a second gate that modulates the channel space charge. As there is a correlation between back-gate voltage and current (ΔI~ΔVg), therefore, the same can be applied to charge on the sensor (ΔI~ΔC). The following relation can be used to correlate the sensor output to the change in surface charge and the gain, which demonstrates the effect of structure.

(13) $$\frac{\mathrm{dI}}{\mathrm{dC}}=\frac{\mathrm{dI}}{{\mathrm{dV}}_{\mathrm{gs}}}\times \frac{{\mathrm{dV}}_{\mathrm{gs}}}{\mathrm{dC}}$$

In Equation (9), the $\frac{\mathrm{dI}}{\mathrm{dVgs}}$ is the gain (gm) or transconductance based on the relation (12). Therefore, the general relation can be simplified to Equation (10).

(14) $$\frac{\mathrm{dI}}{\mathrm{dC}}=g_{m}\times \frac{{\mathrm{dV}}_{\mathrm{bg}}}{\mathrm{dC}}$$

In Equations (13) and (14), the C is the charge sensing on the sensing region.

## 3. Microfluidics and Electrical Packaging

**Figure 3:**
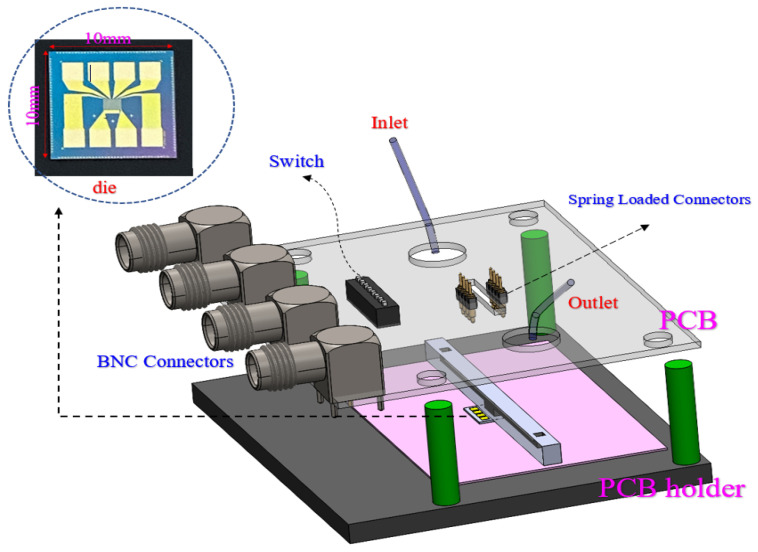
The electrical packaging technique. Spring-loaded connector was used to connect the sensor pads to a switch. The switch is connected to BNC connector.

To prepare the sensor for applying chemical solution, a customized microfluidic was developed that includes a channel running on top of the sensor that encompasses a chamber on the sensing area (see Figure 3). Creating and integrating a microfluidic chip would be extremely difficult if the BioFET worked based on a reference electrode (e.g., Ag/AgCl) inside the solution. However, the OG-JFET structure offers a back gate, making microfluidic integration easier. Developing a microfluidic helps to ensure that the sensor’s pads are not in contact with the solution, as this creates noises in the sensor response and could even damage the sensor.

### 3.1. Electrical Packaging

A spring-loaded connector was used on a PCB to connect the sensor to the semiconductor analyzer (Keithley 4200A-SCS Parameter Analyzer, Tektronix, Beaverton, OR, USA). On the PCB, the spring-loaded connectors were connected to a switch. The switch is used to choose the drain of interest of the sensor. The PCB size is 100 mm × 80 mm, covering a 3D printed structure. Four pillars on the 3D printed design were used to align the PCB with the pads of the sensor. The pads’ area is 1.7 mm × 1.7 mm with 2 mm pitch. The BNC connectors are placed on one side of the PCB. The connection between the PCB and the semiconductor analyzer is established through SMU cables. Before fabricating the fixture, it was modeled in CAD software (solid work) to ensure alignment. A closeup of the electrical packaging is shown in Figure 3.

The die size is 10 mm × 10 mm, and there is a comparatively sizeable total sensing area of 1 mm × 2 mm, contributing 0.7 mm^2^ of the whole die. The chip pad area is generously large, allowing the spring-loaded connector in the electrical packaging scheme.

### 3.2. Microfluidic Packaging

**Figure 4:**
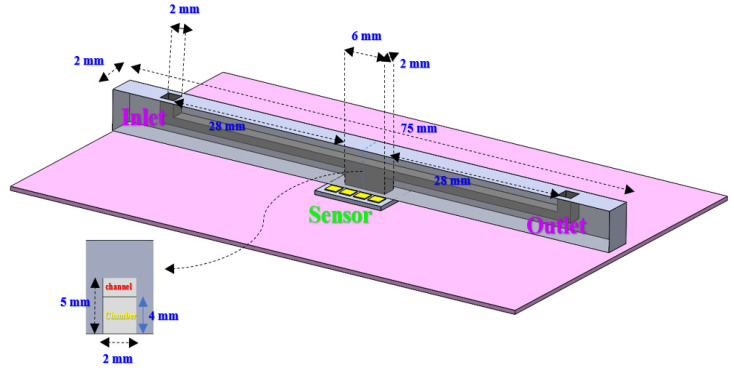
The detailed size and dimensions of microfluidic chip designed to be integrated with OG-JFET. Large sensing area allowed us to create the microfluidic with 3D printing.

The microfluidic channel was fabricated based on the PDMS replica molding method. The mold was 3D printed using the PolyJet method; then, the PDMS was prepared with a 10:1 ratio and poured into the mold for curation. The size and dimensions for the designed microfluidic channel based on the sensor sensing area constraints are shown in Figure 4. To fabricate the microfluidic shown in Figure 4, two separate 2D microfluidics were fabricated. Two parts were then adhered with UV curable glue (MMOBIEL UV LOCA TP-N1000, Overijssel, Netherlands). The adhesion of a PDMS sheet and a microfluidic channel creates a 3D structure placed on top of the sensor.

**Figure 5:**
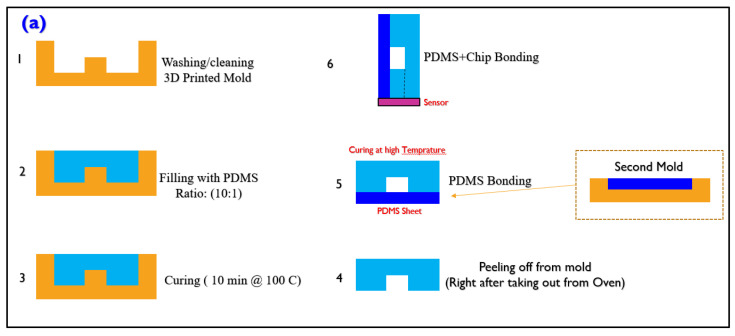

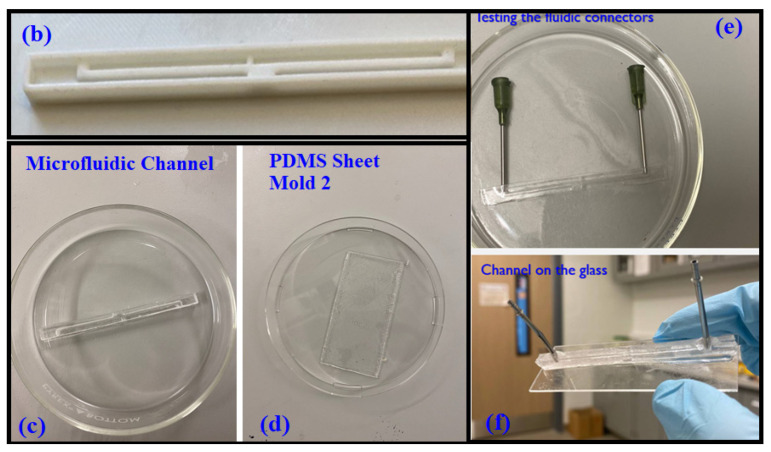
The processes led to the fabrication of a microfluidic channel on top of the sensor. (**a**): the PDMS replica molding process undertaken to create the 3D microfluidic with two 2D PDMS; (**1**) first the 3D printed mold was washed, (**2**) filling the mold with a 10:1 ration of pre-prepared PDMS (after filling the mold, the PDMS and mold were put in the vacuum chamber for 20 mins for degassing), (**3**) after degassing, the PDMS and mold were put in the oven and baked for 5 min at 100 C, (**4**) the PDMS was peeled off from the mold, (**5**) at this point, a channel is put on a PDMS sheet, which has already been prepared with another mold, (**6**) bonding the PDMS and chip. (**b**): the used channel molds. (**c**) showing the peeled-off microfluidic structure. (**d**): the PDMS sheet shown in step 5. (**e**) testing the fluidic connectors with water. (**f**) showing the adhesion of the PDMS channel on the glass.

The microfluidic channel was prepared according to the methods demonstrated in Figure 5. According to our customized protocol of microfluidic fabrication, first, the molds were washed with an ethanol solution to ensure no debris was in the mold. To further improve the washing, the molds were put in the mixture of water and oil for 10 min at 50 °C. Afterward, the molds were washed to be prepared for PDMS injection. The PDMS was prepared with a 10:1 ratio of the base polymer and curing agent, and then the polymer was blended with a stirrer for 10 min at each try. After pouring the PDMS into the mold, it was degassed in the vacuum chamber. The degassing was conducted for 20 min to remove all the bubbles.

The PDMS chip was then placed inside the oven and baked for 5 min at 100 °C. This condition yields a flexible PDMS structure; therefore, it is in excellent condition to easily be peeled off from the thin mold. Other temperatures with a longer time (at lower temperatures) result in a solid PDMS, making it hard to remove, and in most cases, it breaks near the inlet or outlet. At the same time, these processes were tried for another mold, which created a thin layer of PDMS (1 mm) to cover the channel (see Figure 5d). After placing the channel on the PDMS sheet with UV curable glue, the bonding was strengthened by applying UV light on both sides with a hand-held UV lamp (UV 365 nm light) for 5 min. This helped to make a strong bonding for the PDMS–PDMS connection. The mold that includes the channel geometry was placed on a uniform large PDMS sheet; therefore, the issue of the cumbersome bonding process of PDMS–PDMS was avoided. Subsequently, the whole microfluidic channel was cut out under the microscope to release the structure shown in Figure 5f. After removing the structure, it was again heated and immediately put on the chip.

**Figure 6:**
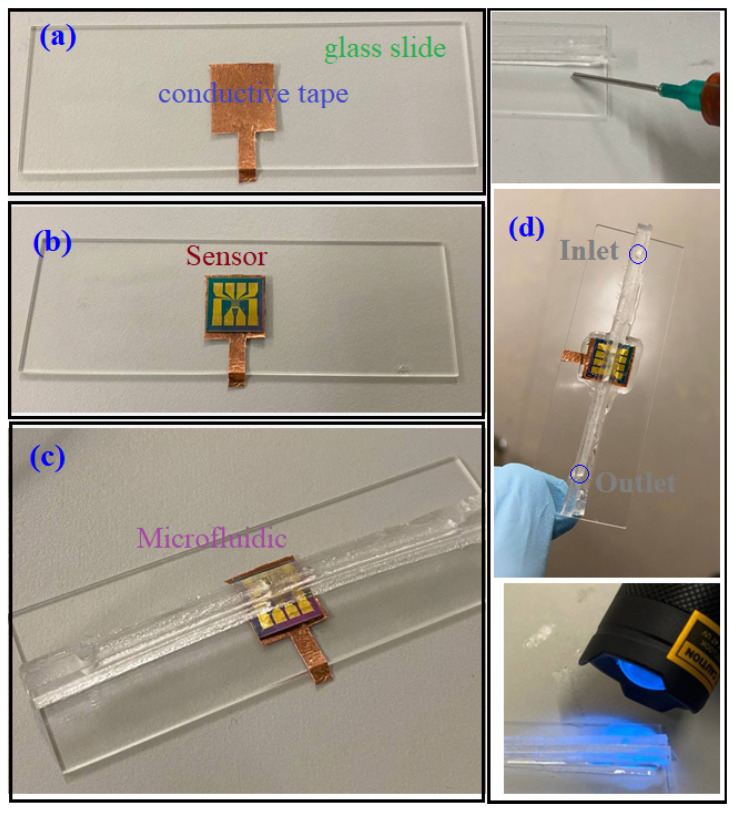
Bonding process of the microfluidic channel on the chip. (**a**): A copper conductive tape was utilized to create the back-gate connection on the glass slide. (**b**): the sensor was put on the sensor. (**c**): after heating the microfluidic, it was aligned with the chip under a microscope. (**d**) filling the gap underneath the microfluidic with UV curable glue and applying UV light to make the structure strongly bonded.

Figure 6 shows the steps for making a back-gate contact connection using the conductive copper tape. The copper conductive tape was soldered to a wire, which connects the wire to a pin header on the PCB as the back-gate connection. It is noteworthy that, as shown in Figure 6, the sensor comes with seven drains, and since they provide almost the same results (transfer characteristics), we arbitrarily used one of them.

### 3.3. Sensor Characterization

**Figure 7:**
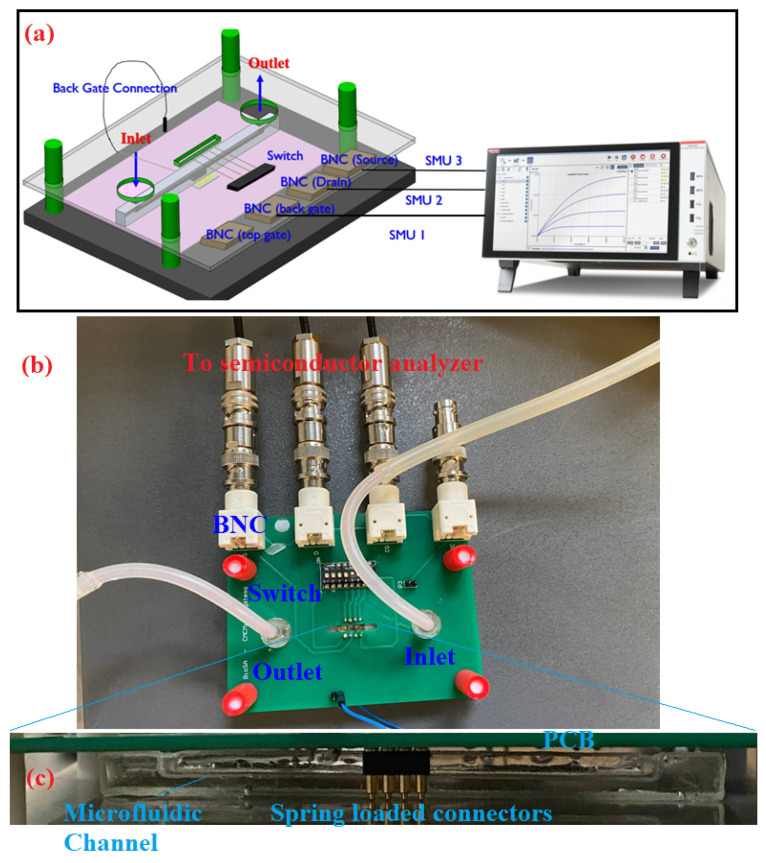
The experiment setup was used to test the OG-JFET sensor. (**a**): shows the CAD design of the fixture used for connecting the sensor to the semiconductor analyzer. (**b**): the fabricated test setup, consisting of PCB (BNC, switch), 3D printed support and microfluidic with its tubing. The back gate was contacted from the back of the sensor to top on the PCB, where it was connected to a BNC connector to the semiconductor analyzer. (**c**): shows the spring-loaded connectors used to connect the OG-JFET pads to the PCB. On the PCB, there is a switch that enables the user to select the specific channel on the OG-JFET sensor.

The sensor was characterized in reverse bias mode by applying a positive voltage to the back gate (see Figure 1) through a semiconductor analyzer (Keithley 4200A-SCS Parameter Analyzer, Tektronix, Beaverton, OR, USA) by a stepwise voltage increment from 0 to 1 V with 0.1 steps. At the same time, the drain–source voltage was swept from 0 to 5 V with 0.2 V increment. The p-n junction was maintained turned off in this mode, ensuring zero current from the back gate into the p-type channel. A general view of the integrated sensor and microfluidic with the fixture is shown in Figure 7. The inlet tube was connected to a syringe, and the solution was loaded. The fluid was maintained stationary inside the channel to ensure the possible noises due to turbulence in the ions flow near the surface. The setup was used for making contact with the sensor pads; therefore, the device could be used without the need for huge probe station. The switch on the PCB made the OG-JFET channel selection much easier. Therefore, when the fluid was in contact with the sensor, the channels could be switched on and off to measure the fluid effects in that area.

## 4. Results

The OG-JFET sensor was fabricated with a standard microfabrication technology (see Figure S1 for more information). Here, the integration with a PDMS-based microfluidic is detailed. The back-gated structure of the sensor allows running a microfluidic channel atop the sensor in the sensing region. In this section, the results associated with the sensor transistor response in both dry mode and when the sensor is exposed to solution in the microfluidic channel are discussed. Furthermore, the results of mathematical modeling and fluidic simulations are explained here.

### 4.1. Sensor Response in Dry Condition

**Figure 8:**
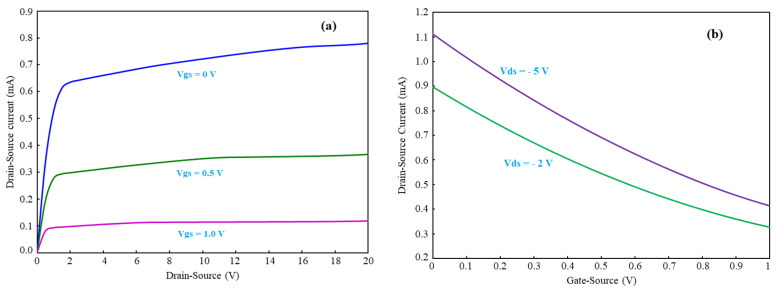
The Ids–Vds and Ids–Vgs curves of OG-JFET. (**a**): Ids vs. drain–source voltage. (**b**): Ids vs. back-gate voltage.

The sensor consists of a p-n junction, which can be imagined as similar to conventional JFET with opened top gate. The top gate is devoted to sensing a solution or any kind of biomaterials. The back gate can be used to control the conduction of the channel. The p-n junction can be turned on or off based on the polarity of the voltages applied on the p-type and n-type layers. If a positive voltage is applied to p-type and a negative to n-type (see Figure 1), the sensor is forward biased, which allows the p-n junction to be opened. The difference between the drain and gate voltage is more than a specific threshold voltage. The calculated turn-on voltage for this p-n junction here is ≈0.67 V. However, when the voltage difference between the drain and back gate reaches this number, the p-n junction is opened, and huge current will be directed from the back gate to the drain, which is not preferable for biosensing applications. For this reason, the sensor is suggested to be used in the reverse-biased condition, as it allows controlling the channel with the back gate for more efficient sensing of the surface charge and solution chemical potential. After being integrated with microfluidics, the sensor was characterized when no solution was on it. The Ids–Vds and Ids–Vgs curves of OG-JFET are demonstrated in Figure 8.

**Figure 9:**
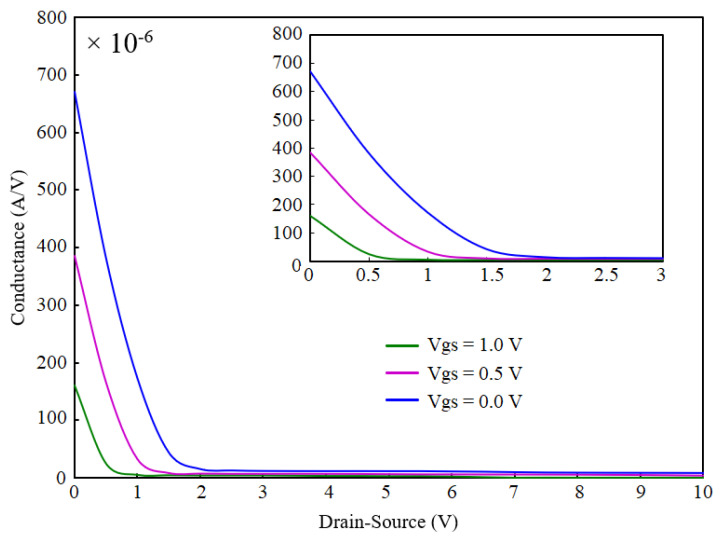
Change of conductance of OG-JFET with back gate.

Figure 9 demonstrates the conductance of OG-JFET for different drain–source voltages when gate source is applied on the sensor. This curve clearly shows the effect of back gate on the control of the channel conductance. According to Figure 9, the conductance decreases when drain–source voltage is applied, narrowing the channel due to back-gate space charge modulation. The channel conductance approaches zero at higher back-gate voltages (=1 V).

**Figure 10:**
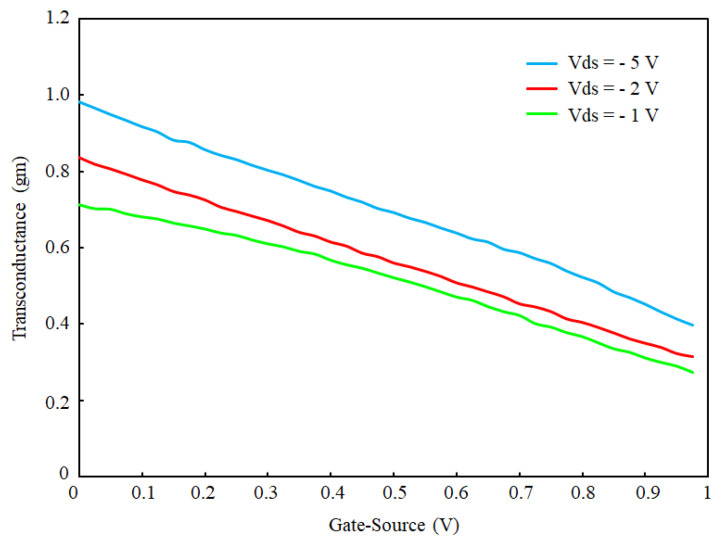
The transconductance of OG-JFET calculated for different values of back-gate voltage.

The transconductance (gm) of the sensor is calculated based on the experimental results (see Figure 10). The gm is depicted vs. back gate that shows the controllability of gain with the back gate. Additionally, the result demonstrates that the gm linearly decreases with increasing the back gate. The gain is calculated by taking the first derivative of the Ids–Vgs curve shown in Figure 8b. The maximum gain of the sensor occurs at the Vgs = 0. It is noteworthy that the Vds enhancement leads to higher gains. The gain estimates the current enhancement in Equations (13) and (14), based on this result.

### 4.2. Solution Tests in Microfluidic Channel

**Figure 11:**
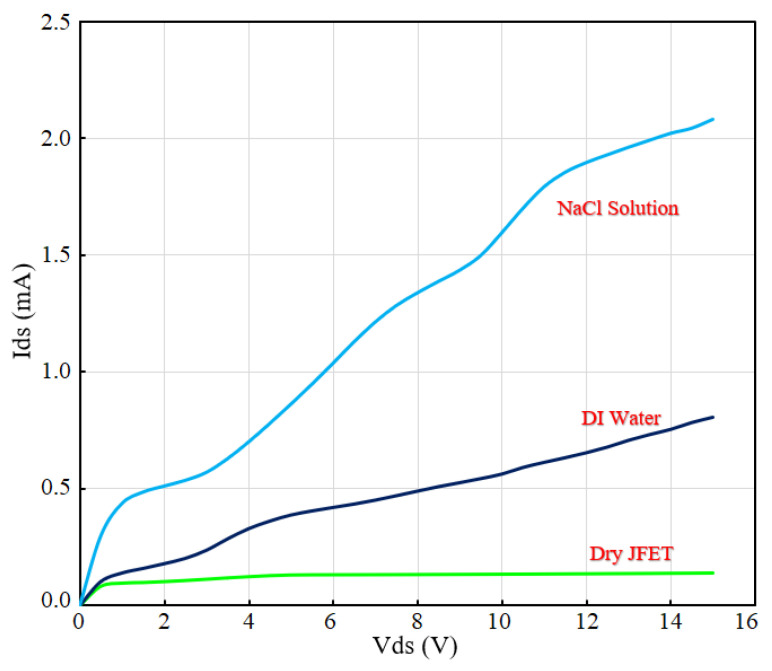
Preliminary test results of sensor with DI water and 0.9% NaCl solution injected in microfluidic channel.

Simple wet tests were performed to demonstrate the fabricated microfluidics functionality and the sensor’s integration. The sensor was tested with DI water and 0.9% NaCl solution to show the sensor’s response to a change in the concentration of ions in the solution. The concentration of ions in the NaCl solution was higher than the DI water, and the sensor showed more current enhancement toward this change in solution (see Figure 11).

Additionally, for precise control over the sensor’s response to change in the surface charge, a solution with different pH was prepared and tested in the microfluidic channel. The fluid was injected into the channel with a syringe pump and then stopped to obtain stationary fluid in the chamber. In our previous work, the change in Vgs with different pH levels was reported to be 39.45 mV/pH, 42.62 mV/pH, 45.79 mV/pH, 48.96 mV/pH, 52.12 mV/pH and 55.29 mV/pH determined for different reference current measurements [62]. These values can be considered as $\frac{{\mathrm{dV}}_{\mathrm{bg}}}{\mathrm{dC}}$ in Equations (13) and (14). The surface charge can be correlated with the pH [62], therefore, we can use pH in this formula. To calculate the above charge (=pH) sensitivities, the back gate electrode of OG-JFET was used, which demonstrates the effectiveness of this sensor topology for measuring surface charge variations without the need for bulky reference electrodes in the solution. Furthermore, the back gate can be used to change the gain of the sensor for changing the current sensitivity.

**Figure 12:**
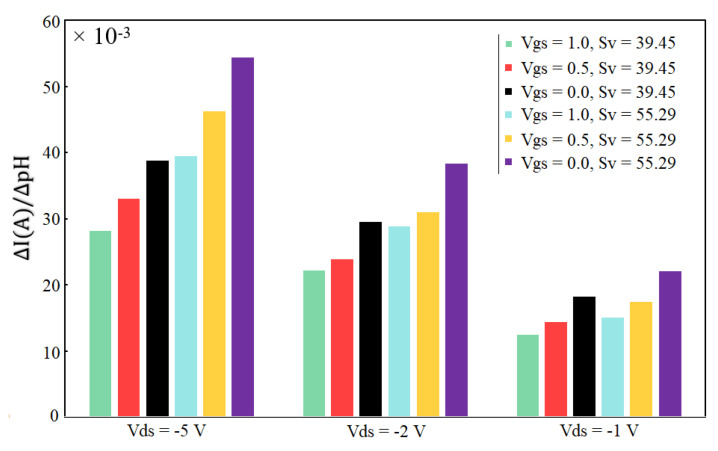
The effect of gain on the current sensitivity for different values of surface charges due to pH variations. Sv is the voltage sensitivity of sensor with unit of mV/pH.

The gain was calculated based on the experimental results using Equations (13) and (14), and used in the calculation of the current sensitivity. Figure 12 shows the effect of back gate on the sensing variation. The back gate in OG-JFET is an advantage in the packaging. It allows easier integration of a microfluidic channel in continuous fluidic; furthermore, it enables changing the working point of sensor and changing the current sensitivity to surface charge.

### 4.3. Mathematical Modeling Results

To confirm the sensor’s transfer characteristics, the mathematical modeling of the sensor is presented in this paper. The mathematical modeling was developed to estimate the sensor response to variation in surface charge. Therefore, the surface charge effect was approximated with a simple model that considered the space charge variation of the p-type channel. The model is simple and cannot be used for general surface potential modeling on the JFET.

**Figure 13:**
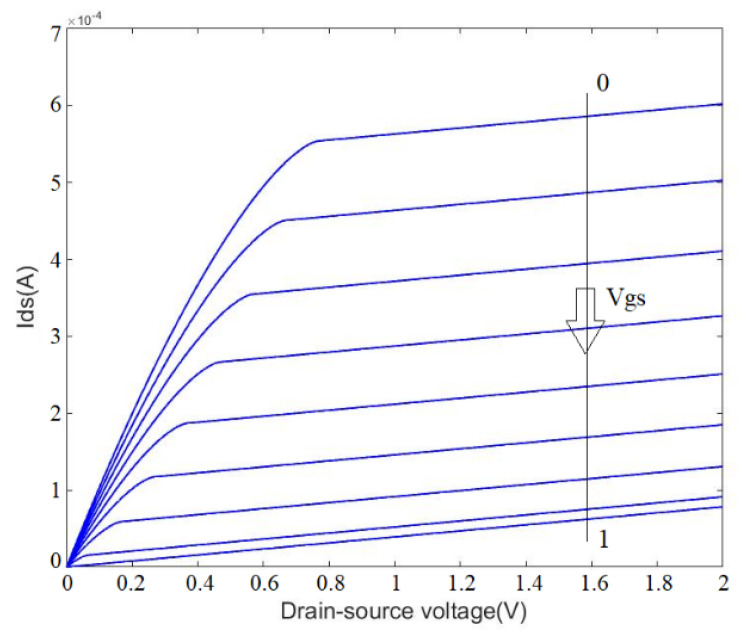
Mathematical results of the Ids–Vds curves for different Vgs values from 0 to 1 with 0.1 steps. The model can predict the sensors response in low values of drain–source voltages before saturation. The back-gate voltage is also limited to the Vpo for good accuracy.

**Figure 14:**
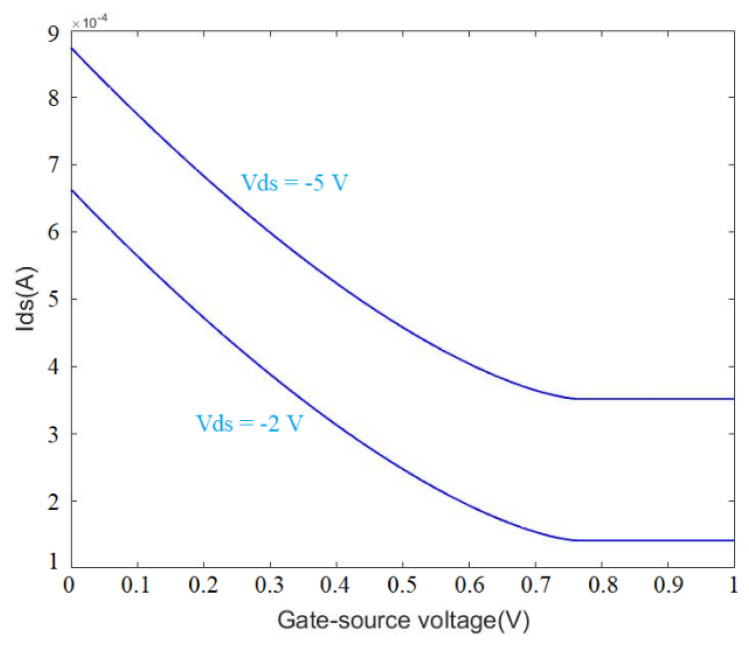
Mathematical modeling result of Ids–Vgs response for Vds values of 2 V and 5 V.

Equation (11) was solved for the gate-source voltage range of 0 to 1 for Vds values 2 V and 5 V (see Figure 13 and Figure 14). A comparison of the mathematical modeling results with the experiment indicates the model was not accurate in predicting the experiment trend for back-gate voltages higher than 0.7 V. After this voltage, the sensor response remained constant, which was not in accordance with the experiments. The mathematical modeling was discussed in previous work [74]; however, the limitations of the model were not discussed for OG-JFET with transfer characteristics being shown. Figure 13 demonstrates the point that the model was not helpful for high back-gate voltage. Therefore, the model introduced in this work and also Ref. [74] can be used for the low back-gate (preferably <0.7) and drain–source voltages (preferably <0.6). A comparison of the experimental result of sensor transfer characteristics (see Figure 8) with model result (see Figure 13) indicates that the non-idealities in fabrication processes, such as non-uniformity of doping levels, uncertainties in controlling the channel length with implantation of source and drain, might be responsible for the inconsistency between the model and the experiment.

**Figure 15:**
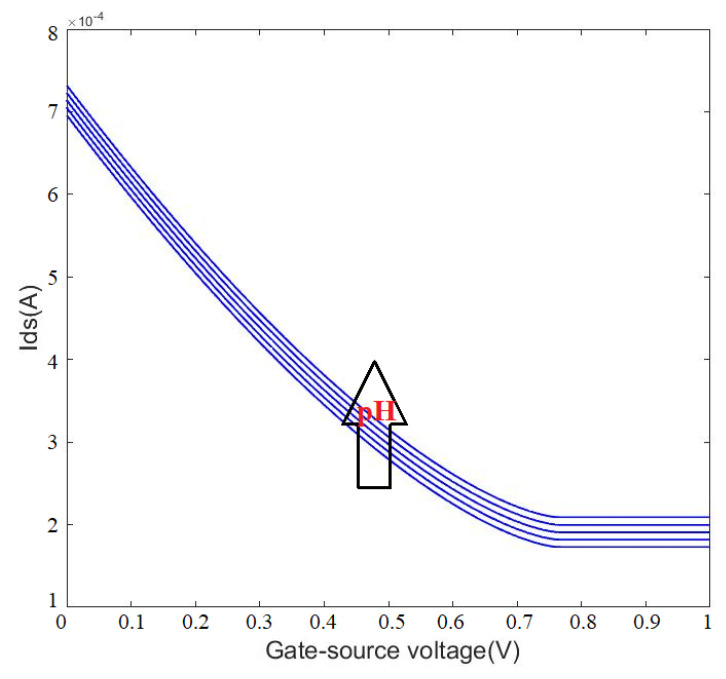
Mathematical modeling result of surface charge effects on the OG-JFET sensing area. The increment in pH results in more negative surface charge.

Figure 15 shows the modeling result of the surface charge effects on the sensor response. The experiments showed that the pH causes an increment in output current. Here, the surface charge modeling was inspired by the work performed in Ref. [75] for NO_2_ detection using a similar JFET structure. However, the charge density was determined using the simulation modeling of pH equivalent surface charge we introduced in our previous work [72]. Same as the experiment and multiphysics simulation, the mathematical model shows current enhancement with an increase in pH (Figure 15).

### 4.4. Microfluidic Simulations

The microfluidic was designed to deliver the sample onto the sensing region of OG-JFET. The fluidic transport inside the channel was analyzed with COMSOL Multiphysics software to gain insight into the fluid dynamics in the chamber atop the sensor and in the channel. The analysis was performed to estimate the fluid velocity in the chamber for different mass flow rates, which is helpful for future design of this kind.

**Figure 16:**
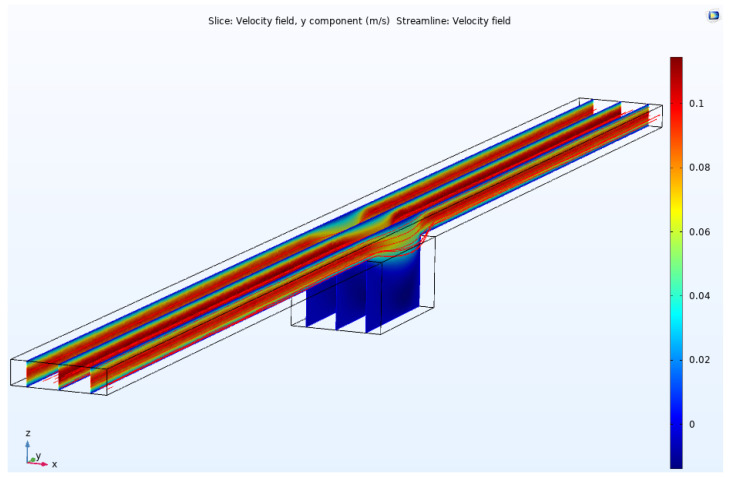
Flow velocity contour in the microfluidic channel for 260 mg/s inlet mass water flow rate.

According to the velocity profile shown in Figure 16, the velocity is higher in the middle of the channel and almost zero near the walls, as the no-slip boundary condition is applied to the channel simulation. When the fluid is approaching the chamber, suddenly, flow velocity decreases, which leads to the transport of fluid partially into the chamber. Therefore, a specific fraction of fluid will always be captured in the chamber in the continuous operation mode of the microfluidic. Considering the sample homogeneity in the base fluid, we can expect the same volume fraction of analyte of interest in the channel.

**Figure 17:**
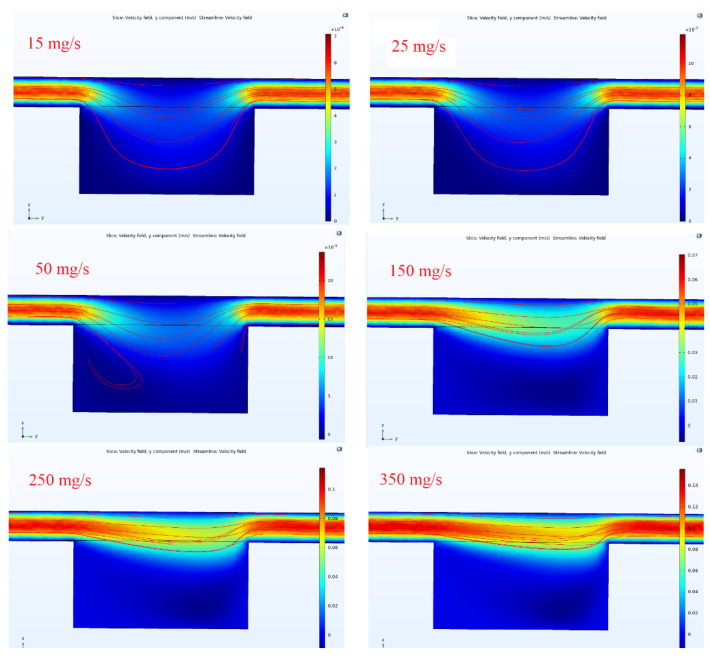
Velocity contours for different values of inlet mass flow rate in the microfluidic channel.

The flow condition is demonstrated by observing the flow with velocity contours in the middle plane of the microfluidic channel. As can be seen in Figure 17, when the flow rate increases from a low value (10 mg/s) to high values (>350 mg/s), the flow starts to become unstable around 50 mg/s and continuous to pass the chamber in the middle. At high flow velocities, the volume fraction of the fluidic trapped in the chamber decreases, as the flow gains high momentum to pass the chamber. For specific applications, such as the cells under perfusion or analyte transportation on the sensing region (with this microfluidic topology), this analysis helps choose the best range of flow rate in the channel.

**Figure 18:**
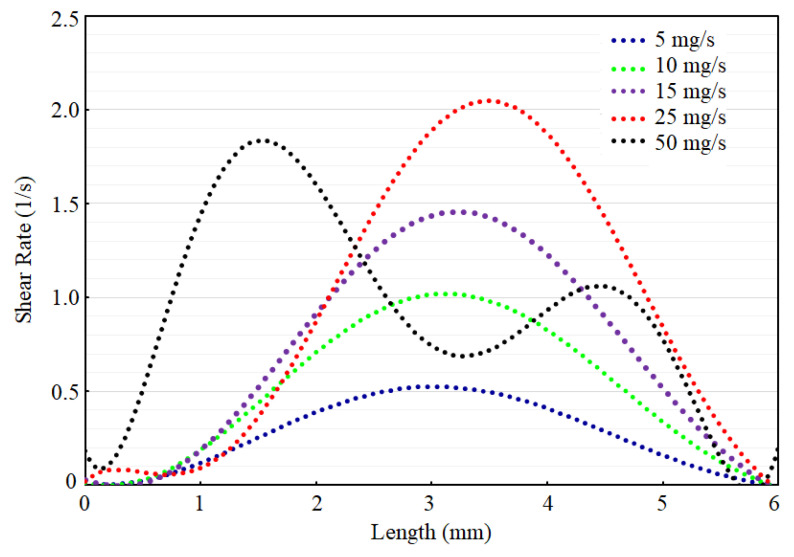
The change in shear rate for different values of inlet mass flow rate condition along the centerline of the chamber.

The flow shear rate distribution is calculated to demonstrate the effect of flow on the sensing region. Shear rate explains the impact of inlet flow on the flow shear stress in the sensing region, which is good for quantifying flow effects on the bottom of the chamber. In other words, the shear rate can be used to demonstrate the effect of change in inlet velocity on the transport at the sensing region. Figure 18 shows the change in shear rate on the centerline of the bottom plane of the chamber where the sensor is placed in the middle.

According to the results shown in Figure 18, the flow is not stable for all ranges of inlet conditions. For instance, the flow around 50 mg/s yields wavy shear stress on the surface, indicating turbulence in the flow, which is not a desirable condition for working a biosensor in continuous mode. Therefore, according to the simulation, we suggest a flow rate lower than 50 mg/s for operation of this sensor in this microfluidic channel. However, to conduct pH tests, we conducted experiments in stationary conditions to avoid stream current in the flow that affects pH sensitivity. The microfluidic analysis provides insights for utilization of this sensor for biosensing applications, which needs the precise control of the injection of an analyte on the sensing region with this chamber-included microfluidics.

## 5. Discussion

Using 3D printed microfluidics, a simple method is introduced in the paper for integration of a miniaturized fluidics part with a FET sensor for life science applications. The methodology allows using two planar microfluidics to create a chamber on top of the sensing area of the sensor. The introduced microfluidic system allows higher feature sizes that make the fabrication feasible with 3D printing. Furthermore, the bonding is achieved through UV curable glue, which makes the integration much easier. Additionally, the design of microfluidics parts avoids the alignment issues mostly associated with small microfluidics that has to be developed in the top area of chip. The microfluidics is testes with simple fluids to show the functionality of sensor and the operation of introduced packaging methodology. It is proved that using the thin microfluidics, as described in this paper, it will be appropriate to be applied on the back-gate FET sensors that are becoming popular due to their enhanced sensing properties. Microfluidics can be used for those sensors to eradicate the need for bulky electrodes in the solution. The introduced packaging method for the FET sensor can be used to create a platform for rapid testing of the solution and avoid the cumbersome process associated with testing the sensor inside the probe station cage that has limited space and is risky due to possible fluid leakages that are detrimental to the expensive machine electronics. Furthermore, it is discussed how the 3D printing (PolyJet) can be recruited to rapidly build a testing fixture integrated with microfluidics for testing a solution and other bioassays on a FET sensor. Additionally, both theoretically and experimentally, it is proved that the back-gate structure can be used to change the gain of the sensor, which enables more sensitivity (e.g., pH sensitivity). For theoretical analysis, a mathematical modeling is introduced, which is useful for prediction of the trends of sensor’s response and comparison with the sensors function. However, more research should be conducted to improve the model and, in particular, the charge modeling in the solution.

The FET sensor in this work is proved to be charge sensitive with a demonstration of simple liquids and pH solutions. Based on the successful utilization of FET sensors in different life science applications, we believe the charge-sensing capability of the current platform could be extended to sensor other charged proteins and chemical reactions that lead to enhancement of the solution’s charge density. Nevertheless, this sensor could be unutilized with receptors for a specific analyte in the solution for other life science applications, since the charge-sensing capability of the sensor enables those sensing mechanisms.

## 6. Conclusions

This research explains the integration of OG-JFET sensor to 3D printed PDMS-based microfluidics for biosensing applications. The microfluidics can be easily fabricated with 3D printed molds. The sensor’s large die size and sensing area (≈0.7 mm^2^) provide a large size, allowing using high-aspect ratios for molding, which makes the PDMS fabrication easier, without the need for complex methods, such as photolithography. The sensor comes with a back-gate structure that enables easier integration with a microfluidic channel without bulky reference electrode integration. Many efforts have been made in the fabrication of miniaturized reference electrodes on the sensing dies, which makes their stability and lifetime a severe matter of concern in integrated microfluidics biosensors. Nevertheless, we have shown in this paper how PolyJET 3D printing technology can be used for the fabrication of PDMS microfluidic molds for a back-gated sensor. The sensor is successfully integrated and tested with a solution to demonstrate its functionality for solution charge sensing, which is the aim of every BioFET sensor.

For this reason, DI water and 0.9% NaCl solution was tested in a microfluidic channel to show the functionality of the integration. To be more precise in demonstrating the effectiveness of back gate and its importance in the integrated sensor, the current sensitivity of the sensor was analyzed, showing the effect of the sensor in controlling the operation point of sensor. Furthermore, it was shown that the back gate can enhance the current sensitivity by the inherent amplification potential that the back gate provides. Put simply, the back gate significantly eases integration by providing an open-gate area for running a microfluidic channel. It can be used to take advantage of the sensor structure to enhance the current sensitivity by changing the gain (gm). It was shown that the change of back gate from 1 V to 0 V changed the gain from ~0.42 mA/V to ~1 mA/V in a constant Vds. It was shown the physically controlled surface charge sensitivity of the sensing layer (here SiO_2_) can be amplified by the sensor’s gain to change the current sensitivity. It was shown that the change of the back gate from 1.0 to 0.0 can increase the sensitivity from ~40 mV/pH to ~55 mV/pH.

## Data Availability

Not applicable.

## Supplementary Materials

The following supporting information can be downloaded at: https://www.mdpi.com/article/10.3390/mi13030425/s1, Figure S1: The fabrication processes of OG-JFET. The fabrication is performed by CMC Microsystem.
