# Supplementary material for: A Hybrid Microfluidic Electronic Sensing Platform for Life Science Applications
Source: Micromachines (Basel). 2022 Mar 10;13(3):425. doi: 10.3390/mi13030425 (PMC8950014; doi:10.3390/mi13030425)
Supplement: Supplementary file 1 [file micromachines-13-00425-s001.zip › micromachines-1615000-supplementary.pdf]

This file explains the fabrication process of OG-JFET sensor. The fabrication process is shown in the Figure S1 and the microfabrication processes are explained.

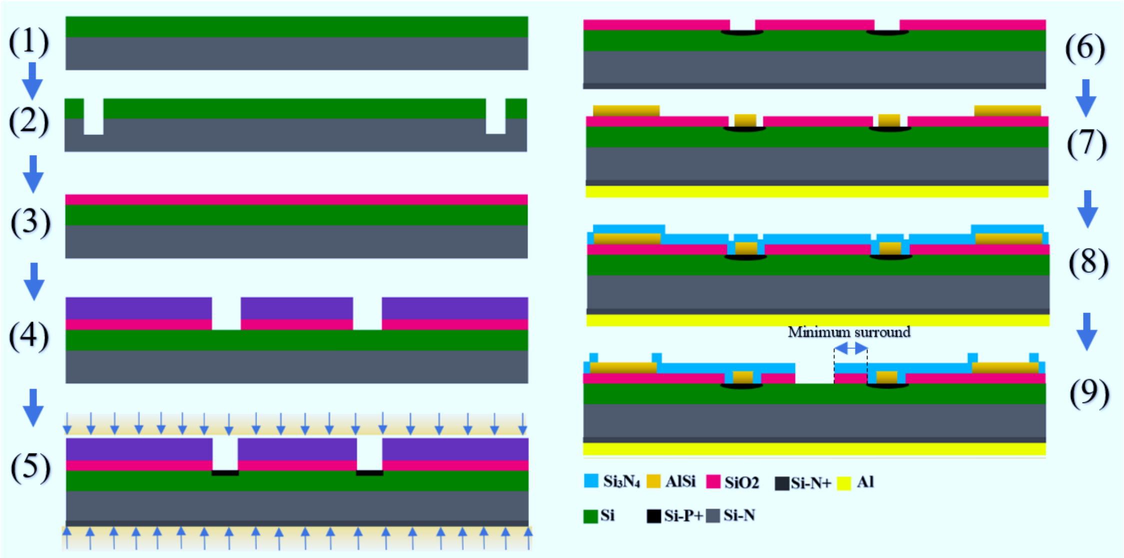

Figure S1. The fabrication processes of OG-JFET. The fabrication is performed by CMC Microsystem

The OG-JFET standard fabrication process according to Figure S1:

(1) Substrate wafer (Silicon <100>, 100 mm diameter, −420 +/−25µm thick, resistivity 0.006 to 0.02 Ohm-cm)

(2) Alignment marker definition (first mask)

(3) PECVD SiO2 deposition

(4) SiO2 etching to Si layer. (second mask)

(5) Front and back-side ion implantation

(6) implantation annealing

(7) front and back side metallization and annealing

(8) PECVD Si3N4deposition (passivation layer)

(9) Pad and channel opening.
